# Overexpression of *AtLOV1* in Switchgrass Alters Plant Architecture, Lignin Content, and Flowering Time

**DOI:** 10.1371/journal.pone.0047399

**Published:** 2012-12-26

**Authors:** Bin Xu, Noppadon Sathitsuksanoh, Yuhong Tang, Michael K. Udvardi, Ji-Yi Zhang, Zhengxing Shen, Maria Balota, Kim Harich, Percival Y.-H. Zhang, Bingyu Zhao

**Affiliations:** 1 Department of Horticulture, Virginia Tech, Blacksburg, Virginia, United States of America; 2 Department of Biological Systems Engineering, Virginia Tech, Blacksburg, Virginia, United States of America; 3 Plant Biology Division, Samuel Roberts Noble Foundation, Ardmore, Oklahoma, United States of America; 4 Department of Plant Pathology, Plant Physiology and Weed Science, Virginia Tech, Blacksburg, Virginia, United States of America; 5 Department of Biochemistry, Virginia Tech, Blacksburg, Virginia, United States of America; 6 BESC – The BioEnergy Science Center of U.S. Department of Energy, Ardmore, Oklahoma, United States of America; University of Massachusetts Amherst, United States of America

## Abstract

**Background:**

Switchgrass (*Panicum virgatum* L.) is a prime candidate crop for biofuel feedstock production in the United States. As it is a self-incompatible polyploid perennial species, breeding elite and stable switchgrass cultivars with traditional breeding methods is very challenging. Translational genomics may contribute significantly to the genetic improvement of switchgrass, especially for the incorporation of elite traits that are absent in natural switchgrass populations.

**Methodology/Principal Findings:**

In this study, we constitutively expressed an *Arabidopsis* NAC transcriptional factor gene, *LONG VEGETATIVE PHASE ONE* (*AtLOV1*), in switchgrass. Overexpression of *AtLOV1* in switchgrass caused the plants to have a smaller leaf angle by changing the morphology and organization of epidermal cells in the leaf collar region. Also, overexpression of *AtLOV1* altered the lignin content and the monolignol composition of cell walls, and caused delayed flowering time. Global gene-expression analysis of the transgenic plants revealed an array of responding genes with predicted functions in plant development, cell wall biosynthesis, and flowering.

**Conclusions/Significance:**

To our knowledge, this is the first report of a single ectopically expressed transcription factor altering the leaf angle, cell wall composition, and flowering time of switchgrass, therefore demonstrating the potential advantage of translational genomics for the genetic improvement of this crop.

## Introduction

Switchgrass (*Panicum virgatum* L.), a native North American perennial C4 grass with great natural diversity and adaptability over a large portion of the continent, is a prime candidate biomass crop for the US [1]. The genetic improvement of switchgrass for large scale biomass feedstock production started only recently. Currently, most switchgrass cultivars are synthetic, having been selected directly from their original habitats, and average biomass yields range from four to ten tons/acre per year depending on cultivar and field conditions [2]. Due to the polyploid genome and gametophytic self-incompatibility of switchgrass, breeding the crop is a significant challenge [3].

As the study of switchgrass at the molecular level is relatively new, researchers can benefit from the knowledge gained through the extensive study of model plant species, such as *Arabidopsis*, rice, *Medicago truncatula*, and *Brachypodium distachyon* [4]–[6]. Candidate genes isolated from model plant species could also be directly expressed in crop plants, using efficient tissue culture-based genetic transformation systems, to improve agronomically important traits [7]. In certain cases, novel desirable phenotypes could be triggered by the ectopic expression of a candidate gene in different plant species [8]. The overexpression of a transcription factor identified from another plant species, especially the potential master regulator of a given signaling pathway, may disturb the biological and metabolic processes of switchgrass and generate extreme or novel phenotypes. Analysis of the gene expression profile of the transgenic genotype may contribute to the functional genomics of switchgrass by associating gene functions with certain phenotypes.

An erect leaf phenotype can improve plant architecture by decreasing shade effects, thereby maximizing the plant biomass yield of a dense field population [9]. Under dense planting conditions, a higher leaf area index (the ratio of the upper leaf surface area to land area) could increase light interception for photosynthesis, and small leaf angles against vertical stems (erect leaves) are required to obtain a high leaf area index [10]. Increasingly small leaf angles have been associated with the higher yields of recently developed rice cultivars [11]. Mutations in several brassinosteroid (BR)-related genes, the overexpression of an *ARGONAUTE* gene (*OsAGO4*), and a mutation in a *KANADI* gene (*SLL1*) have been shown to induce smaller leaf angles in rice plants [12]–[15]. The altered leaf angles could be the result of developmental changes in the leaf collar region, as in the case of several BR-related mutants [12], or could be caused by leaf rolling (upward curved leaf), as in the case of *OsAGO4* and *SLL1* mutants [14], [15]. In switchgrass, a linear relationship between the leaf area index and seasonal biomass yield has been observed [16].

The accumulation of aboveground biomass declines after plants begin flowering [17]. Therefore, plants with delayed flowering time have extended vegetative growth and usually produce more biomass. In switchgrass, lowland ecotypes flower late in high latitude areas, producing higher yields than upland ecotypes [2]. Although the genetic components controlling switchgrass flowering time have not been characterized, it is hypothesized that they are similar to those of maize because both switchgrass and maize belong to the same subfamily of the PACCAD clade (consisting of the Panicoid, Arundinoid, Chloridoid, Centothecoid, Aristidoid, and Danthonioid lineages) [18], [19]. Interestingly, maize flowering time, unlike that of *Arabidopsis* or rice, is primarily controlled by a collection of quantitative loci with smaller effects [20], although some conserved flowering genes, such as *FLOWERING LOCUS T* (*FT*, *ZCN8* in maize) and *INDETERMINATE1* (*ID1*), have also been identified in the maize genome [21], [22]. The possibility of controlling switchgrass flowering time by identifying and manipulating its flowering genes is an attractive one.

Lignin is a phenolic polymer derived from three monolignols [hydroxyphenyl (H), guaiacyl (G), and syringyl (S)] via combinatorial radical coupling reactions [23]. In lignocellulosic bioenergy research, reducing the lignin content of switchgrass feedstock is a major improvement objective for several reasons: the high lignin content of switchgrass cell walls prevents hydrolytic enzymes from accessing the polysaccharides, absorbs these hydrolytic enzymes, and inhibits the activities of the hydrolytic and fermentation enzymes used in the biological biofuel conversion process [24]. The lignin biosynthesis pathways are controlled by at least ten key enzymes that are evolutionarily conserved across angiosperms [24], [25]. Several transcription factors directly regulating monolignol biosynthetic genes have also been identified in a number of model plant species [8], [26]–[28]. Although most orthologous genes involved in monolignol biosynthesis pathways can be identified from the switchgrass EST database and the functions of several lignin genes have been validated in switchgrass, our knowledge of switchgrass lignin biosynthesis is still limited [29]–[33]. The identification and characterization of switchgrass genotypes with differing lignin contents may contribute to the detailed dissection of monolignol pathways.

Transcription factor proteins bind to conserved *cis*-elements and either transactivate or suppress the expression of groups of genes involved in multiple cellular processes [34]. The expression of plant transcription factors is controlled by developmental cues and/or environmental stimuli [35]. Therefore, the knockout of a transcription factor often results in pleiotropic effects. Conversely, the constitutive expression of a transcription factor may lead to gain-of-function traits. NAC (NAM, ATAF1/2 and CUC2) domain-containing genes constitute one of largest plant-specific transcription factor families, regulating biotic/abiotic stress responses, photoperiod, and plant development [36]. Transgenic plants with desirable agronomically important traits have been obtained by the manipulation of NAC transcription factors in various plant species [37]–[40]. It was recently reported that the overexpression of a NAC domain transcription factor gene, *Long Vegetative Phase 1* (*AtLOV1*, At2g02450), contributes to cold tolerance and delayed flowering time under long day photoperiod conditions in *Arabidopsis* [41]. AtLOV1 functions within the photoperiod pathway in which its overexpression negatively regulates the expression of a *CONSTANS* gene (*CO*) and results in a late-flowering phenotype. Interestingly, the overexpression of AtLOV1 also regulates cold response genes such as *COLD-REGULATED 15A* (*COR15A*) and *COLD INDUCED 1* (*KIN1*) to generate cold tolerance in the transgenic lines. Consequently, AtLOV1 has been identified as a master regulator of both flowering and cold response pathways in *Arabidopsis* [41].

For perennial plants such as switchgrass, enhancing cold tolerance could improve stand establishment and survivability, and delaying flowering time could lead to plants with higher overall biomass. However, delaying flowering time may negatively impact switchgrass survivability over the winter if it causes plants to remain vegetative through the onset of killing frosts [42], [43]. Therefore, it is desirable to introduce both late flowering and cold tolerance phenotypes for switchgrass improvement.

In this paper, we report that switchgrass plants with ectopic overexpression of *AtLOV1* have delayed flowering time, erect leaves, and increased lignin content; however, they do not have improved cold tolerance. We performed microarray analysis of the *AtLOV1* transgenic plants, and putative genes involved in flowering control, cell wall biosynthesis and organ development were identified among the 104 genes regulated by *AtLOV1*. Breeding the erect leaves and delayed flowering traits conditioned by the *AtLOV1* transgene into elite switchgrass cultivars might improve switchgrass biomass feedstock production, especially under densely planted field conditions.

## Materials and Methods

### Gene Cloning and Switchgrass Genetic Transformation

A Gateway compatible binary vector pVT1629 [30] modified from pCAMBIA1305.2, in which a *ccd*B(B) cassette could be replaced with the gene of interest through an LR reaction (Invitrogen, Carlsbad, CA), was developed for switchgrass genetic transformation. The maize *Ubi1* promoter [44] was used in the vector to drive the expression of the transgene.

The full length *AtLOV1* gene was amplified from the genomic DNA of the *Arabidopsis thaliana* ecotype Columbia-0 using the primers LOV1_BamHFor and LOV1_SalRev (Table S1). The PCR product was cloned into the vector pENTR/D-TOPO® (Invitrogen), and the *AtLOV1* gene sequence was confirmed by sequencing. The *AtLOV1* gene was then sub-cloned into the expression vector, pVT1629 [45], with an LR cloning kit (Invitrogen), according to the user's manual. The resultant pVT1629-AtLOV1 vector was transformed into the *Agrobacterium tumefaciens* strain C58C1 through electroporation. Somatic embryogenic calli induced from the seeds of a selected switchgrass line, HR8, were used for genetic transformation [46]. The regenerated plants were selected using 50 mg/L of hygromycin B (Sigma Chemical Co., St. Louis, MO). Regenerated plants from independent calli were further verified by PCR, GUS staining (data not shown) and Southern blot analysis. More detailed switchgrass tissue culture and transformation methods have been described previously [30], [46]. Six independent transgenic lines were transplanted into Miracle-Gro Potting Mix (Miracle-Gro Lawn Products, Marysville, OH) in 1.1×10^−2^ m^3^ pots in the Horticulture greenhouse at Virginia Tech (Blacksburg, VA), with temperatures set at 28°C/day and 22°C/night and 12–14 h of light. Soil water content was maintained at ∼80%. Wild type (WT) plants regenerated from non-transformed calli were also grown under the same conditions. Because switchgrass is self-crossing incompatible, the progeny of T_0_ “escapes” were used as WT plants for comparison with the transgenic T_0_ plants. T_1_ generation transgenic plants were obtained by crossing independent T_0_ transgenic plants with the WT controls. The presence or absence of the *AtLOV1* transgene in the T_1_ plants was detected by PCR using the primers LOV1_BamHFor and LOV1_SalRev (data not shown). T_1_ plants without the transgene were designated as the WT plants for comparison with the various phenotypes of transgenic T_1_ plants. T_1_ plants were maintained in the greenhouse under the same conditions as previously described.

### RT-PCR and Southern Blot

Total RNA was extracted from the leaves of transgenic and WT plants using the TRIzol® Reagent (Invitrogen). cDNA was generated by reverse transcription with SuperScript III (Invitrogen). The expression of *AtLOV1* was detected by RT-PCR using LOV1.2_FOR and LOV1_SalRev (Table S1). Genomic DNA was extracted from four T_0_ transgenic *AtLOV1* plants for Southern blot analysis for which a DNA fragment of the *HPTII* gene was used as a probe. Approximately 10 µg of switchgrass genomic DNA was digested with the restriction enzyme *Hin*dIII, which is absent in the *HPTII* gene. The digested DNA samples were electrophoresed in a 0.8% agarose gel and then transferred to a nylon membrane (Whatman Schleicher and Schuell, Keene, NH). A detailed Southern blot method has been previously described [46].

### Lignin Content and Monolignol Composition Measurement

Four-month old plants grown in the greenhouse were used for lignin content analysis. Tillers with emerged panicles were chosen for sampling of the stem and leaf tissue. The stems of the first node, including a 2-cm-long leaf base and a 2-cm-long leaf sheath close to the leaf collar of each plant, were sampled for lignin content analysis. Plant tissues were collected from three wild type controls and three independent T_0_ transgenic lines. The tissue samples were dried at 60°C in an oven and ground using a coffee grinder; a detailed method for this procedure has been previously described [30]. Lignin and ash contents were measured according to the standard biomass protocol developed by the National Renewable Energy Laboratory (NREL) [47]. Monolignol composition was analyzed with a recently modified thioacidolysis protocol [48]. The silylated sample was injected into the GC column (Restek RTX5-MS, 1 µM film thickness, 30 M ×3.2 mM i.d., Thames Restek UK Ltd., Windsor, UK), and the GC-MS was performed on a VG 70SE double focusing magnetic sector instrument interfaced to an HP5790 GC. The results were analyzed according to a previously reported method [30].

### Plant Growth Measurement and Statistical Analysis

T_1_ generation plants segregating either with or without the *AtLOV1* transgene were used for plant growth measurements. The growth conditions of the T_1_ plants are described above. Flowering time and biomass yields were recorded from nine transgenic and nine WT plants grown in the greenhouse and harvested at the same time in mid-September of 2010.

In each experiment, at least three biological and technical repeats for each plant sample were conducted for statistical analysis. The comparison of treatment means was conducted by the Tukey HSD and Tukey-Kramer (for lignin contents) multiple comparison procedures using JMP software version 7 (SAS Inc., Cary NC).

### Microarray Analysis

WT control and *AtLOV1* transgenic plant materials were collected from newly emerged tillers when they had two fully collared leaves. Total RNA was extracted from the joint region between the leaf and the leaf sheath, including a 2-cm-long leaf base and a 2-cm-long leaf sheath close to the leaf collar, using TRIzol Reagent (Invitrogen) and was purified by Qiagen RNeasy columns (QIAGEN, Valencia, CA). To minimize the effect of the heterogeneous genetic background of switchgrass, we selected three independent T_1_ transgenic lines for gene expression analysis. Each T_1_ line was propagated in three pots by transplanting split tillers of similar growth stage. Therefore, each pool was taken from three clonal plants of one distinct T_1_ line, and three pools were taken from each of three distinct T_1_ lines. Nine transgenic plants in total, divided into three replications, were used for this experiment. Plant tissues from three independent T_1_ lines were pooled for RNA extraction. The nine transgenic plants were used to produce three RNA samples, which were considered as three biological replicates. As a control, three wild type plants were also split into nine pots, and the pooled tissues from these plants were used to isolate three RNA samples. RNA was quantified and evaluated for purity using a Nanodrop Spectrophotometer ND-1000 (NanoDrop Technologies, Wilmington, DE) and a Bioanalyzer 2100 (Agilent, Santa Clara, CA).

Three pooled RNA samples, each from three independent T_1_ plants, were used as biological replicates for the microarray. For each sample, 500 ng of total RNA was used for expression analysis on the custom-designed switchgrass cDNA chip Pvi_cDNAa520831 (Affymetrix, Santa Clara, CA). Probe labeling using the IVT Express Kit, chip hybridization and scanning were performed according to the manufacturer's instructions (Affymetrix). Data normalization between chips was conducted using the Robust Multichip Average (RMA) [49]. Gene selections, based on the Associative T-test [50], were made using Matlab software (MathWorks, Natick, MA); this analysis method has been detailed in a previous report [51]. To reduce the family-wide false positive rate, we used a Bonferroni-corrected P-value threshold of 4.07977E-07. This value was derived from 0.05/N [52], where N is the number of probes sets (122,556) on the chip. For hierarchical clustering, the z-scores for the expression values of selected genes were calculated and imported into MeV open software (http://www.tm4.org/mev/). Pearson's coefficient was used to calculate the similarity in expression patterns between genes.

The DNA sequences of the microarray probes that detected genes with altered expressions were used as queries for BLAST searching against the Expressed Sequence Tag (EST) databases of both NCBI (National Center for Biotechnology Information) and the Switchgrass Functional Genomics Server hosted at the Samuel Roberts Noble Foundation (http://switchgrassgenomics.noble.org/) (updated in June 2011), as well as the switchgrass genome sequence database (http://www.phytozome.net/panicumvirgatum.php). Probe sets that showed similar expression patterns, as well as those that yielded the same hit to the database, were merged. A few probes detected genes of viral origin, possibly due to viral contamination of the plants. After the elimination of the repeated probes and the probes against virus sequences, a total of 104 switchgrass genes were shown to have altered expression levels between the WT and transgenic lines. The longest ESTs or probe target sequences of the 104 switchgrass hits were used to perform BLAST searches against the Genbank database (http://blast.ncbi.nlm.nih.gov/Blast.cgi). The maize, rice and *Arabidopsis* gene annotations were used as the basis for the manual annotation of the switchgrass genes.

### Real-time qRT-PCR

The microarray data were validated with real-time RT-PCR (qRT-PCR) on ten selected genes with two biological repeats. The RNA samples were extracted from the pooled T_1_ transgenic and wild type control plants as described above in the microarray analysis section. qRT-PCR was performed with the ABsolute^TM^ Blue QPCR SYBR® Green plus ROX mix kit (Thermo Fisher Scientific, Inc., Waltham, MA) using the ABI 7500 Real-Time PCR System. The PCR reaction was performed in a 25 µl reaction volume following the manufacturer's instructions. Each sample had three technical replicates, and the data were normalized against the expression of the reference gene, switchgrass *Actin2*. The dissociation curves showed that the primers used for qRT-PCR were gene-specific. The qPCR experiments were repeated two times, yielding similar results in each repetition. Only one set of data is presented in this study. The primers used for qRT-PCR are listed in Table S2.

## Results

### Overexpression of *AtLOV1* in switchgrass caused an erect leaf phenotype

**Figure 1:**
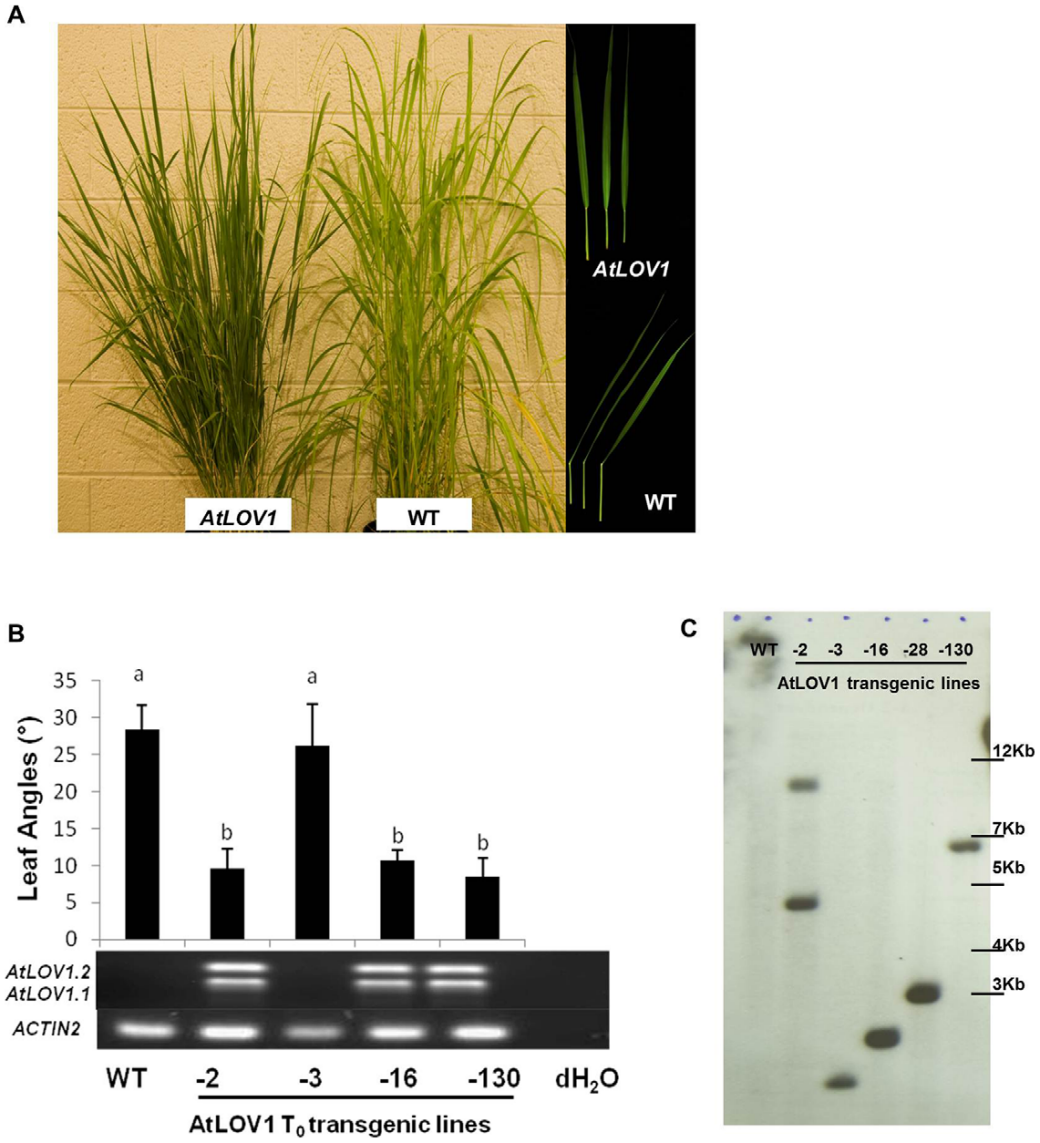
Overexpression of *AtLOV1* caused erect leaf phenotype in switchgrass. (**A**) Transgenic plants had smaller leaf angles compared to wild type (WT). (**B**) The leaf angles of four independent T_0_ transgenic lines were associated with the expression of *AtLOV1* in plants as detected by RT-PCR (noting that *AtLOV1* pre-mRNA could be alternatively spliced in switchgrass, yielding the transcripts *AtLOV1.1* and *AtLOV1.2*). Different letters above the bars indicate statistically significant differences (α = 0.01). (**C**) Southern blot analysis with an *hph* probe showed T-DNA integration in five T_0_ transgenic lines.

The full-length *Arabidopsis AtLOV1* gene containing its original introns was transformed into switchgrass, and expression of this transgene was driven by the maize *Ubiquitin 1* promoter [44]. The presence of T-DNAs in the genomes of the transgenic plants was analyzed by PCR amplification with primers specific to the *AtLov1* gene (data not shown). We selected six putative T_0_ transgenic lines to be acclimated to the greenhouse for further analysis. Most *AtLOV1* transgenic lines had smaller leaf angles than the WT control plants. The small leaf angles made these plants more erect (Figure 1A). The erect leaf phenotype of transgenic plants was associated with the expression of *AtLOV1* (Figure 1B). The *AtLOV1* gene can be transcribed into two transcripts (*AtLOV1.1* and *AtLOV1.2*) in *Arabidopsis* via alternative splicing (Figure S1), and both transcripts encode proteins that can be localized into the plant nucleus (Figure S3). Interestingly, when *AtLOV1* was ectopically expressed in switchgrass, the gene was also able to be spliced into two transcripts (Figure 1B). The identities of the two transcripts were confirmed by cloning and sequencing (data not shown). The integration and copy numbers of T-DNAs in the plant genomes were further confirmed by Southern blot analysis. The transgenic lines carry one to two copies of the T-DNA insertions (Figure 1C).

To further analyze the phenotypes of the *AtLOV1* transgenic switchgrass plants, we generated T_1_ plants by crossing two independent T_0_ *AtLOV1* transgenic plants (lines −16 and −130) with WT plants. The T_1_ plants segregated with either erect or non-erect leaf phenotypes in a 1∶1 ratio, and the erect leaf phenotype co-segregated with the presence of the *AtLOV1* transgene, providing further confirmation that *AtLOV1* caused the erect leaf phenotype in switchgrass (data not shown).

### Alterations of the abaxial:adaxial collar height resulted in erect leaves

**Figure 2:**
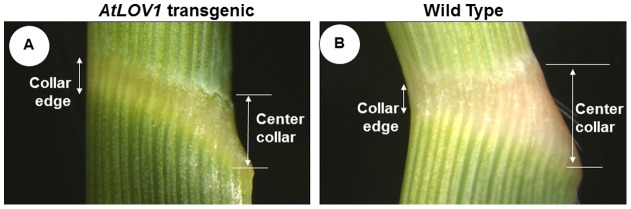
Plant morphology at the leaf collar region in *AtLOV1* transgenic and wild type (WT) plants. A side view of the leaf collar region of transgenic (**A)** and WT plants (**B**). Compared to WT plants, *AtLOV1* transgenic plants have increased length of the collar edge region but decreased length of the center collar region.

The leaf phenotype of transgenic plants was investigated by visually examining the leaf collar (or lamina joint) region. As shown in Figure 2 and Table 1, in comparison with WT plants, *AtLOV1* transgenic plants had an increased edge-collar height but a decreased center-collar height. The alteration of the abaxial:adaxial collar height ratio resulted in smaller leaf angles in *AtLOV1* transgenic switchgrass plants and yielded an erect leaf phenotype (Figure 1A).

**Table 1:** Means (n = 9) for the length of the leaf collar region of *AtLOV1* transgenic and wild type control plants.

T_1_ segregated plants	Length of collar edge (mm)	Length of collar center (mm)	Ratio between lengths of collar edge and collar center
***AtLOV1***	2.1 (±0.1) ^a**^	2.0 (±0.1)^ **^	1.04 (±0.01)^ **^
**Wild Type**	3.2 (±0.2)	1.3 (±0.3)	2.65 (±0.24)

a SE (standard error) is in parentheses. ** Statistically significant differences (p<0.01) between wild type and transgenic plants.

### Lignin content and monolignol composition of cell wall biomass were altered in *AtLOV1* transgenic plants

Greater lignin content in leaf and stem tissue could increase the mechanical strength of plants, thus contributing to an erect leaf phenotype [53]. In this report, the T_0_ plants were investigated for lignin content. We first assayed the lignin deposition in the stems of T_0_ transgenic plants using a phloroglucinol stain [54]. The stems of transgenic plants stained more darkly than WT plants (Figure S2), suggesting that the transgenic plants contained more 4-O-linked hydroxycinnamyl aldehydes, which are related to lignin content. To confirm the phloroglucinol staining result, we quantitatively measured the lignin content and monolignol composition of three independent transgenic lines by GC-MS. As summarized in Table 2, the transgenic plants had approximately 6–10% greater total lignin content than the WT control from the pooled biomass of three WT plants. Moreover, the transgenic plants had an altered monolignol composition with an increased S:G ratio (Table 2).

**Table 2:** Means for the total lignin content and monolignol composition of three independently transformed T_0_ *AtLOV1* transgenic plants compared to the averaged data from three wild type plants.

Switchgrass T_0_ Lines	Total lignin (mg/100 mg)	Monolignol composition (%)			S:G
		Hydroxyphenyl (H)	Guaiacyl (G)	Syringyl (S)	
**Wild Type**	20.6 (±0.2)^a**^	1.2 (±1.0)^**^	60.4 (±3.4)^**^	38.4 (±2.4)	0.64 (±0.01)^**^
***AtLOV1*** **-2**	21.8 (±0.3)	3.9 (±0.6)	51.7 (±4.5)	44.4 (±4.0)	0.86 (±0.01)
***AtLOV1*** **-16**	21.8 (±0.1)	n/a	n/a	n/a	n/a
***AtLOV1*** **-130**	22.7 (±0.2)	5.2 (±1.2)	51.8 (±1.3)	43.0 (1.7±)	0.83 (±0.01)

The biomass of three wild type plants was pooled together as one sample for lignin content analysis.

a SE is in parentheses. ** Statistically significant differences (p<0.01) between wild type and transgenic plants.

n/a not available.

### *AtLOV1* transgenic switchgrass plants had delayed flowering time but not increased biomass yield under greenhouse conditions

Nine independent T_1_ transgenic plants and nine WT plants were evaluated for growth under greenhouse conditions. Panicle emergence (first visible heads appearing from the leaf sheaths, considered to be the start of flowering) of the transgenic lines was approximately 4–6 days later than that of the WT controls. In maize, the timing of the floral transition is measured by counting the leaf number, and delayed flowering time can result in plants with more leaves [22]. Therefore, we adapted a similar methodology to compare flowering times among switchgrass plants. After approximately 4 months of growth in the greenhouse, the number of tillers that had flowered and their total leaf number were counted for both transgenic and WT plants. As shown in Table 3, *AtLOV1* transgenic switchgrass plants had fewer flowering tillers, and there were 10% more leaves on the flowering tillers, indicating that the overexpression of *AtLOV1* delayed flowering time.

**Table 3:** Mean number of tillers that had flowered at two stages of development and the number of leaves on flowered tillers for *AtLOV1* transgenic plants compared to wild type plants.

T_1_ plants	Number of plants	Flowered tillers, ES (%)^a^	Flowered tillers, LS (%)^b^	Leaf number of flowered tillers, LS
***AtLOV1***	9	1.8 (±1.8)^ **^	42.7 (±5.1) ^c^	6.3 (±0.1) ^**^
**Wild Type**	9	20.3 (±4.0)	48.6 (±3.6)	5.7 (±0.1)

The means were based on two independent segregating T_1_ progenies from single-insertion transgenic plants.

a ES: early flowering stage, three months after planting. ^b^ LS: late flowering stage, four months after planting. ^c^ SE is in parentheses. ** Statistically significant differences (p<0.01) between wild type and transgenic plants.

The transgenic and WT plants were harvested in mid-September of 2010 after approximately 4 months of growth in the greenhouse. Compared to WT plants, the transgenic plants had similar total tiller number and tiller stem width but slightly reduced height (Table 4). Individual transgenic plants had slightly less aboveground biomass but similar belowground and whole plant biomass (Table 4). Therefore, the *AtLOV1* transgenic switchgrass plants grown in pots under greenhouse conditions had delayed flowering time but not increased biomass yield in comparison with the WT.

**Table 4:** *AtLOV1* transgenic plants have uncompromised whole plant biomass yield but slightly decreased aboveground plant biomass yield.

T_1_ Plants	Number of plants	Tiller number	Plant height (cm)	Stem width (mm)	Biomass yields (g)		
					AP biomass	BP biomass	WP biomass
**WT**	9	38.1 (±2.4) a	146.8 (±1.0)^**^	5.0 (±0.1)	140.9 (±3.6)^**^	98.5 (±7.8)	239.4 (±10.8)
***AtLOV1***	9	37.0 (±2.1)	138.0 (±1.2)	4.8 (±0.1)	111.6 (±3.8)	104.4 (±6.1)	216 (±8.2)

AP, aboveground plant; BP, belowground plant; and WP, whole plant.

a SE is in parentheses. ** Statistically significant differences (p<0.01) between wild type and transgenic plants.

### Global gene expression analysis of *AtLOV1* transgenic switchgrass

**Figure 3:**
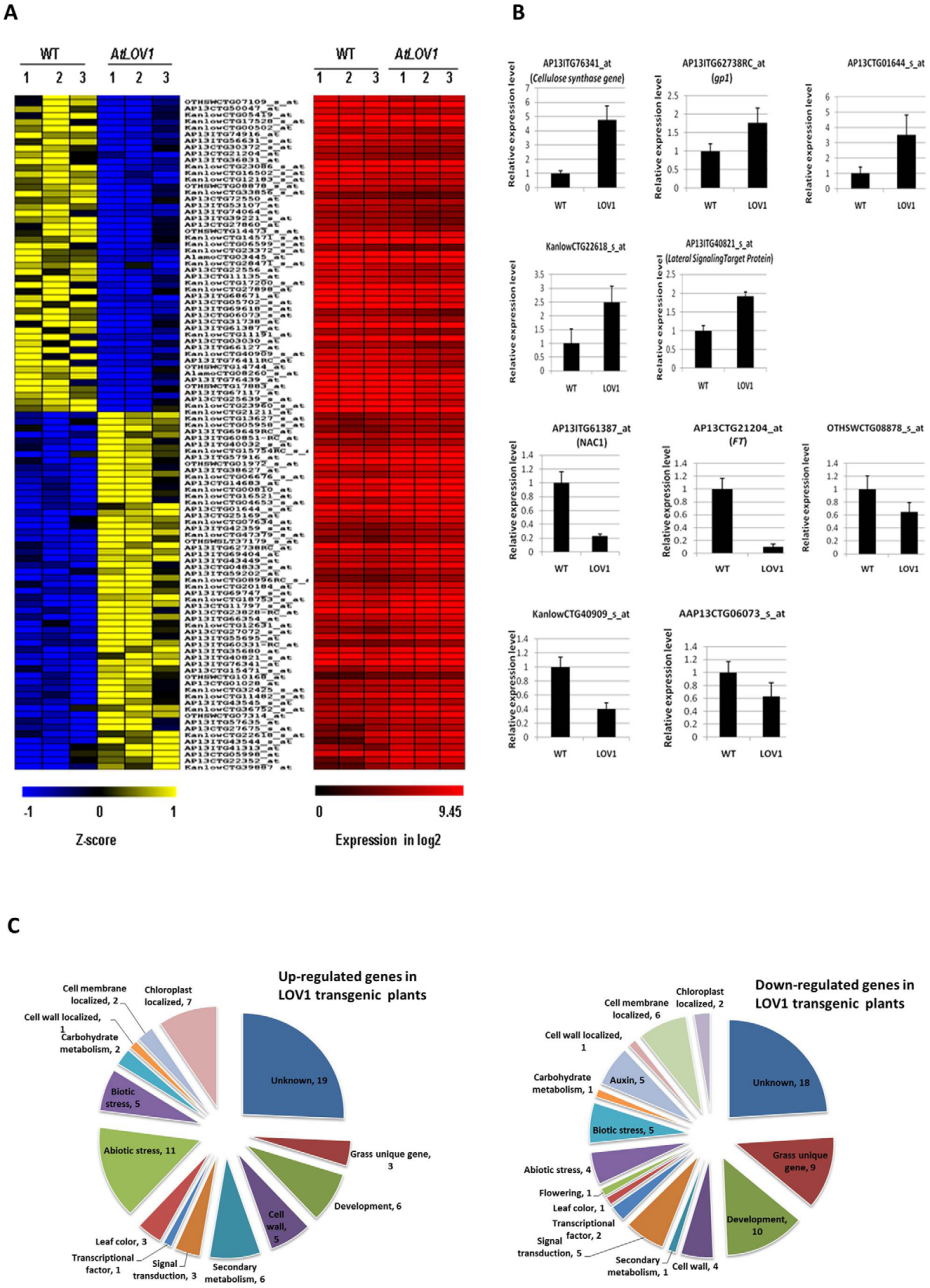
Microarray analyses of differentially expressed genes in *AtLOV1* transgenic plants. (**A**) Heat map of 104 differentially expressed genes showing that 55 genes were significantly up-regulated and 49 genes down-regulated greater than two-fold in transgenic plants. (**B**) Expression levels of ten selected genes were validated with real time-qPCR; the detected genes were indicated by their microarray probe ID, with corresponding orthologous gene names in parentheses when available. (**C**) The differentially expressed genes were annotated and classified. Three biological repeats of the microarray and two biological repeats of the qPCR were conducted.

To explore possible molecular mechanisms and to identify the switchgrass genes associated with the phenotypes of *AtLOV1* transgenic plants, we performed global gene expression profiling using an Affymetrix switchgrass cDNA chip (Figure 3). The reliability of the microarray assay was validated by qRT-PCR on ten selected genes (Figure 3B). The overexpression of *AtLOV1* induced the altered expression of 104 switchgrass genes, among which 55 were up-regulated and 49 down-regulated, with more than two-fold expression changes (Figure 3C). Among the 104 genes, 37 genes encoded unknown proteins. The other 67 switchgrass genes were manually annotated for their putative biological functions according to their rice or *Arabidopsis* homologs. Because the whole genome sequence of switchgrass is not available, the available switchgrass EST sequences had limited power to identify putative gene functions during manual annotation, and most genes were classified as having unknown function (Figure 3C). Nevertheless, our results indicated that the overexpression of *AtLOV1* changed the expression of genes involved in general development and cell wall biosynthesis (Figure 3C and Table S3).

Through this global gene expression analysis, we identified genes potentially related to cell wall biosynthesis (e.g., KanlowCTG40909_s_at, microarray probe IDs), cell fate determination (e.g., AP13ITG40821_s_at), and flowering control (e.g., AP13CTG21204_at). However, we did not identify any known abaxial-adaxial cell fate-determining genes (e.g., *KANADI* family genes or BR-synthesis and signaling pathway genes) that could directly explain the erect leaf phenotype. The potential functional roles of several of these genes are discussed below.

## Discussion

### *AtLOV1* regulates flowering time in both *Arabidopsis* and switchgrass

Previous reports suggested that the overexpression of *AtLOV1* delayed flowering time and enhanced cold tolerance in *Arabidopsis* [41]. In switchgrass breeding programs, the selection of cultivars with a delayed flowering phenotype is desirable because such cultivars may produce more biomass due to their extended vegetative growth. However, a prolonged growing period may compromise the winter survivability of these cultivars, especially if they are grown more than one hardiness zone north of their native range [42], [55]. Increased cold tolerance can improve the winter hardiness of lowland cultivars, which are sensitive to low temperatures in winter [43], [56]. Therefore, the switchgrass homologs of *AtLOV1* may provide an opportunity for the genetic improvement of switchgrass germplasm. NAC transcription factors, including *AtLOV1*, have a conserved N-terminal NAC domain and a variable C-terminal transcriptional activation region (TAR) [36]. We BLAST searched against the current switchgrass EST database with the full-length NAC or TAR sequences of *AtLOV1*; no sequences with significant similarity to *AtLOV1* were identified in the switchgrass EST database. We also BLAST searched the rice and maize EST and genomic DNA databases, which allowed us to identify several AtLOV1 homologs. However, none of these genes have been previously characterized. Therefore, we are not sure which homologs are the actual orthologs of *AtLOV1*. Because we failed to identify a switchgrass ortholog of *AtLOV1*, we attempted to express *AtLOV1* directly in switchgrass. Our results demonstrated that *AtLOV1* transgenic switchgrass had delayed flowering, as has been described for *Arabidopsis* [41]. In *Arabidopsis*, the overexpression of *AtLOV1* negatively regulated the expression of *FT* and resulted in a late-flowering phenotype [41]. Our microarray and real time RT-PCR analyses also identified a gene homologous to the rice and *Arabidopsis FT* gene [57] that was down-regulated by the expression of *AtLOV1* in switchgrass (microarray probe ID: AP13CTG21204_at; corresponding EST GeneBank No. HO298851.1) (Figure 3B). The down-regulation of this *FT*-like gene may contribute to the delayed flowering time phenotype in switchgrass, although the functions of the *FT*-like gene in switchgrass germplasm have not been characterized. Switchgrass flowering time, similarly to that of maize, is regulated by both photoperiod-dependent and autonomous pathways [22], [42], [58], [59]. Therefore, the switchgrass *FT* homolog identified in our microarray analysis may have similar functions to the maize *FT gene* [22]. Direct manipulation of the *FT*-like gene may result in delayed flowering time in switchgrass.

The overexpression of *AtLOV1* increases cold tolerance in *Arabidopsis* [41]. However, in this study, we failed to identify switchgrass genes with obvious homology to known cold-related genes through microarray analysis. The *AtLov1* transgenic plants were not obviously more cold resistant than the control plant (data not shown). It is possible that AtLOV1 regulates cold-related genes in *Arabidopsis* but not in switchgrass [41].

### *AtLOV1* produces two alternatively spliced forms in both *Arabidopsis* and switchgrass

The *AtLOV1* gene has been shown to be alternatively spliced, generating two transcript forms (ANAC034/035, or LOV1.1/1.2) in both *Arabidopsis* and switchgrass, and both of these forms are localized to the nucleus (Figure S3). The phenotypes associated with *AtLOV1* in *Arabidopsis* have been ascribed mainly to the longer transcript (*AtLOV1.2*) [41]. However, in our study, we used genomic DNA of the *AtLOV1* gene, capable of producing both transcripts, to transform the switchgrass (Figure 1B). Therefore, it is unclear if the phenotypes of the transgenic switchgrass plants were caused by the longer or shorter transcript of *AtLOV1*.

### Ectopic expression of *AtLOV1* confers novel phenotypes in switchgrass

One of the largest families of plant-specific transcription factors [36], NAC proteins have been found to be key regulators of plant development and stress perception [60]. In this study, the overexpression of an *Arabidopsis* NAC gene *AtLOV1* in switchgrass resulted in smaller leaf angles and increased lignin content. However, such phenotypes were not previously reported for the overexpression of *AtLOV1* or any other NAC transcription factor in *Arabidopsis* [15], [41], [61], [62]. Therefore, it is unclear if these phenotypes were due to the influence of *AtLOV1* on the same biological functions that it alters in *Arabidopsis*. The transfer of *Arabidopsis* transcription factor genes into crop plant species could result in unexpected phenotypes. For example, transgenic rice plants overexpressing the *Arabidopsis SHN1* gene showed greater cellulose and less lignin content in their cell wall biomass [8], whereas in *Arabidopsis*, *SHN1* was only reported to regulate the epicuticular wax content of leaf surfaces [63]. Despite the fact that the novel phenotypes of transgenic switchgrass or rice plants may not reflect the biological functions of the genes targeted in *Arabidopsis*, the improved agronomic traits generated by transformation have significant applications in the genetic improvement of crop plants.

The genes regulated by *AtLOV1* in switchgrass could be revealed through the analysis of conserved *cis*-elements in the promoters of the switchgrass genes identified in our microarray analysis (Supplemental data, Table S3) [36]. Direct manipulation of these targeted genes may generate desirable phenotypes useful to the breeding of switchgrass for biomass production. However, without a fully sequenced switchgrass reference genome, it is difficult to identify *cis*-elements at this time.

We used switchgrass microarrays for the global gene expression analysis of transgenic switchgrass plants. Because the erect leaf phenotype was most dramatic in the leaf and leaf collar regions, RNA was extracted from the leaf base, leaf collar and leaf sheath regions for the microarray analysis. Our results showed significantly altered expression of 104 switchgrass genes in response to the expression of *AtLOV1* (supplemental data, Table 3).

The microarray analysis identified 16 differentially expressed genes that are putatively related to plant development (Figure 3C). One up-regulated gene product (probe ID: AP13ITG40821_s_at; corresponding to EST No. HO250279.1) is homologous to the human *Lateral Signaling Target Protein*, which serves as a negative regulator of epidermal cell growth factor [64]. However, the functions of plant homologs of this gene have not yet been characterized in any plant species. It will be interesting to study this switchgrass gene further and determine if it has a similar role in the regulation of leaf collar cells development.

Rice mutants with reduced brassinosteroid (BR) content or reduced BR-sensitivity were often severely dwarfed but had erect and dark green leaves [10], [12], [65]–[67]. Interestingly, *AtLOV1* transgenic switchgrass also had erect and dark green leaves (data not shown), and the heights of these plants were slightly shorter than those of the WT controls. However, global gene expression analysis did not identify any genes significantly homologous to known members of BR-related pathways (Table S3), suggesting either that an unidentified BR-independent genetic pathway contributed to the erect leaf phenotype or that *AtLOV1* directly targeted genes downstream of BR signaling that control leaf angle.

Leaf curling also impacts leaf angles, often via changes in leaf abaxial and/or adaxial cell fates [15]. In *Arabidopsis*, the density of abaxial epidermal cells is regulated by a group of genes within the *YABBY* and *KANADI* families [68]–[70], while the adaxial cell identity is controlled by genes belonging to the HD-ZIP III family (e.g., *PHB, PHV*) [69]. Maize and rice *KANADI* family genes also function in defining the abaxial cell identity [15], [71]. However, the leaves of *AtLOV1* transgenic switchgrass lines were not severely curved (data not shown), suggesting that leaf curling was not a major contributing factor to the small leaf angle of *AtLOV1* transgenic switchgrass. No genes homologous to those involved in abaxial or adaxial cell fate determination were identified in the microarray analysis.

In this study, we demonstrated that the overexpression of *AtLOV1* in switchgrass slightly increased the total lignin content of cell wall biomass (Table 2). As a self-incompatible plant species, synthetic switchgrass cultivars have heterozygous genetic backgrounds, and most phenotypes are still segregating in a population of a given switchgrass cultivar. Therefore, it is difficult to find a perfect wild type control to compare phenotypically with transgenic plants, as every individual transgenic line has a slightly different genetic background. In this study, we attempted to measure the lignin content of a wild type control by pooling plant tissue from three individual wild plants, which we consider representative of the averaged lignin content of a switchgrass cultivar. We identified multiple independent *AtLOV1* transgenic lines with slightly higher lignin contents than the averaged data of wild type control plants, suggesting that this lignin content increase was caused by the overexpression of *AtLOV1*. However, this conclusion should be further validated by measuring the lignin contents of T_1_ progenies derived from a single plant that are segregating of the transgene.

Although low lignin content is preferred for the biological conversion of lignocellulosic switchgrass feedstock to biofuel [72], biomass feedstock with high energy content and low mineral residues is highly desirable for other bioenergy production processes, such as pyrolysis and combustion [73]. Through microarray analysis, we identified a number of differentially expressed genes involved in phenylpropanoid metabolism that include nine genes related to cell wall biosynthesis; among these genes, five were up-regulated and four were down-regulated. One up-regulated gene (probe ID: AP13ITG62738RC_at; EST No. FL891887.1) showed significant homology to the *GP1* that is involved in plant cell wall extension and biosynthesis [74]. Another up-regulated gene (probe ID: AP13ITG76341_at; EST No. FL759004.1) encoded a protein with a conserved cellulose synthase domain, although it was not clustered with other cellulose synthases in dicot species (data not shown). The biological functions of most switchgrass genes have not yet been characterized; therefore, we currently have no evidence if the gene (EST No. FL759004.1) actually encodes a functional cellulose enzyme and how it might contribute to the regulation of cell wall composition in *AtLOV1* transgenic switchgrass plants. Nevertheless, the candidate genes identified in this study could be directly manipulated in to the hope of altering the cell wall composition of switchgrass.

An erect leaf phenotype has been observed to contribute to greater biomass yield because it improves plant architecture through increased shade avoidance, maximizing plant biomass yield in a dense field population [10], [11]. In this report, *AtLOV1* plants had an erect phenotype; however, there was no significant difference between transgenic and non-transgenic switchgrass plants in terms of overall biomass yield (Table 4). However, we were only able to measure the biomass yield of individual *AtLOV1* transgenic switchgrass plants grown in pots under greenhouse conditions, and it will be interesting to determine if *AtLOV1* plants actually produce more biomass under high planting density in the field. The expression of *AtLOV1* slightly reduced plant height, which may have partially contributed to the slight decrease of the aboveground biomass yield (Table 4). It is also possible that the overexpression of a transgene has a “penalty” for the transgenic plants, which could be triggered by any number of factors. By backcrossing the transgenic crop plants to wild type control plants, we may identify switchgrass plants with an optimum level of transgene expression that only carry valuable agronomic traits [75].

### Global gene expression analysis of the *AtLOV1* transgenic switchgrass plants

In this study, we identified switchgrass genes regulated by the overexpression of *AtLOV1* through microarray analysis. Further characterization of genes regulated by *AtLOV1* may help us unravel the genetic pathways underlying small leaf angle, cell wall biosynthesis, and flowering time. However, because only RNA transcript differences are detected by microarray analysis, the relationships between the genetic regulation and actual levels of active proteins for these pathways are unknown. Switchgrass is a polyploid plant with a large and complex genome, making it a difficult system in which to conduct reverse genetics studies [55]. Ectopic overexpression of certain transcription factors could trigger novel phenotypes with improved agronomic performance in switchgrass. Expression profiling of the transformed genotypes provides a valuable tool for functional genomics in switchgrass [8]. The identification and characterization of the switchgrass genes regulated by these transcription factors may support a molecular basis for efficient and sustainable biomass production.

In summary, we demonstrated that the overexpression of *AtLOV1* in switchgrass delayed flowering time, decreased leaf angle, and increased lignin content in the biomass. Expression profiling of the transgenic plants identified a set of candidate switchgrass genes associated with the novel phenotypes. These candidate genes could be targets of genetic engineering and molecular breeding in switchgrass. Under greenhouse conditions, the overall biomass yield of transgenic switchgrass was not significantly different from that of non-transgenic plants; however, the *AtLOV1* plants may show an improvement over the wild type due to their erect leaves and delayed flowering phenotypes if they are grown under high-density field conditions. This study highlights the potential of translational genomics as an approach to the molecular breeding of switchgrass.

## Supporting Information

Figure S1 ***AtLOV1*** **has two transcripts due to alternative splicing.** ***AtLOV1.1*** **has four exons and three introns, whereas** ***AtLOV1.2*** **has three exons and two introns.** The filled boxes represent exons, and the lines represent introns. The numbers indicate the nucleotide position.
(TIF)

Figure S2 **Phloroglucinol staining of the lignin deposition patterns in** ***AtLOV1*** **transgenic and wild type (WT) plants.** The darker red stain in *AtLOV1* suggests it has a higher lignin content than the WT control plant.
(TIF)

Figure S3 **The subcellular localization of AtLOV1.1 and AtLOV1.2 fused with C-terminal GFP proteins indicates that both localized to the plant nucleus.** (**A**) GFP control, (**B**) AtLOV1.1:GFP, (**C**) AtLOV1.2:GFP. The GFP signal was observed under a fluorescence microscope (400 x). The green foci in (**B**) and (**C**) represent the plant nuclei. The bars represent 50 µm.
(TIF)

Materials and Methods S1 (DOC)

References S1 (DOCX)

Table S1 **Primers used for PCR and RT-PCR.**
(DOCX)

Table S2 **Primers used in qRT-PCR for the validation of ten selected genes identified by microarray analysis.**
(DOCX)

Table S3 **Differentially expressed genes in** ***AtLOV1*** **transgenic switchgrass.** | | | |, no information available; NA, homologs cannot be identified.
(DOC)
