# Supplementary figures and images for: Overexpression of AtLOV1 in Switchgrass Alters Plant Architecture, Lignin Content, and Flowering Time
Source: PLoS One. 2012 Dec 26;7(12):e47399. doi: 10.1371/journal.pone.0047399 (PMC3530547; doi:10.1371/journal.pone.0047399)

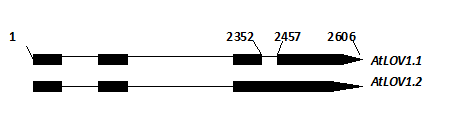

Supplement: Figure S1 — AtLOV1 has two transcripts due to alternative splicing. AtLOV1.1 has four exons and three introns, whereas AtLOV1.2 has three exons and two introns. The filled boxes represent exons, and the lines represent introns. The numbers indicate the nucleotide position. (TIF) [file pone.0047399.s001.tif]

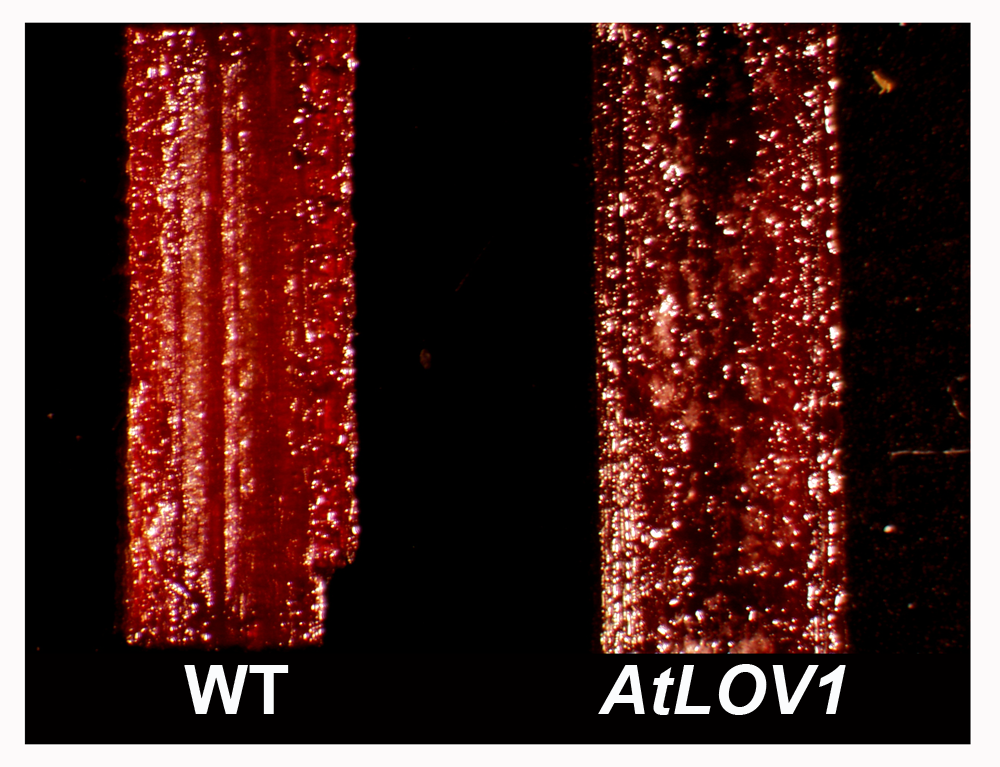

Supplement: Figure S2 — Phloroglucinol staining of the lignin deposition patterns in AtLOV1 transgenic and wild type (WT) plants. The darker red stain in AtLOV1 suggests it has a higher lignin content than the WT control plant. (TIF) [file pone.0047399.s002.tif]

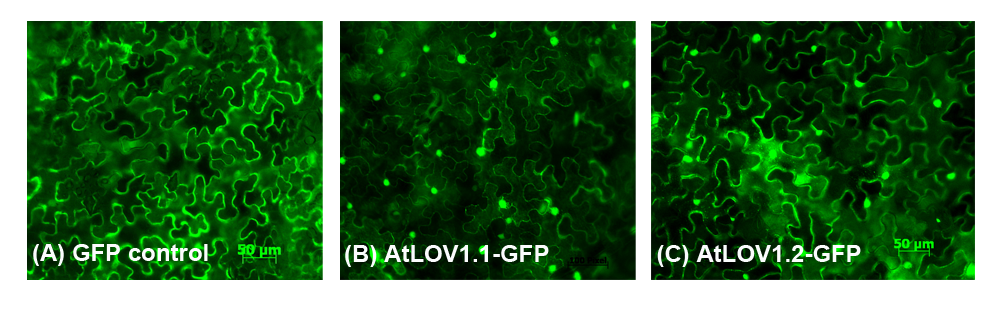

Supplement: Figure S3 — The subcellular localization of AtLOV1.1 and AtLOV1.2 fused with C-terminal GFP proteins indicates that both localized to the plant nucleus. (A) GFP control, (B) AtLOV1.1:GFP, (C) AtLOV1.2:GFP. The GFP signal was observed under a fluorescence microscope (400 x). The green foci in (B) and (C) represent the plant nuclei. The bars represent 50 µm. (TIF) [file pone.0047399.s003.tif]
