# Supplementary material for: Overexpression of AtLOV1 in Switchgrass Alters Plant Architecture, Lignin Content, and Flowering Time
Source: PLoS One. 2012 Dec 26;7(12):e47399. doi: 10.1371/journal.pone.0047399 (PMC3530547; doi:10.1371/journal.pone.0047399)
Supplement: Materials and Methods S1 — (DOC) [file pone.0047399.s004.doc]

**Supplementary Materials and Methods**

**Cellular Localization of AtLOV1.1 and AtLOV1.2**

The two transcripts of *AtLOV1*, *AtLOV1.1* and *AtLOV1.2*, were amplified from cDNA of Arabidopsis ecotype Columbia-0, cloned into pENTR/D vector and sequenced. The genes were subcloned into pEarlyGate101 by LR reaction (Invitrogen, Carlsbad, CA). The targeted genes cloned in pEarlyGate101 is driven by 35S promoter and fused with a C-terminal GFP gene . The resultant binary vectors were electro-transformed into *Agrobacterium* strain GV3101. A transient expression assay was conducted to observe the subcellular localization of AtLOV1.1:GFP, AtLOV1.2:GFP and the control GFP by injecting the *Agrobacterium* strain carrying corresponding vectors into *Nicotiana benthamiana* leaf according to a protocol described previously . The GFP signal was observed under an Axio Observer A1 florescent microscope (Carl Zeiss MicroImaging LLC, Thornwood, NY).

**Histology and Microscopy**

The internodes of *AtLOV1* transgenic and wild type control plants were stained with phloroglucinol reagents (Pomar *et al.*, 2002) to analyze the lignin deposition patterns and visualized under an Olympus SZXZ-RFL3 diagnostic microscope (Olympus America, Melville, NY, USA). Detailed protocol was described previously .
