# Supplementary material for: Overexpression of AtLOV1 in Switchgrass Alters Plant Architecture, Lignin Content, and Flowering Time
Source: PLoS One. 2012 Dec 26;7(12):e47399. doi: 10.1371/journal.pone.0047399 (PMC3530547; doi:10.1371/journal.pone.0047399)
Supplement: References S1 — (DOCX) [file pone.0047399.s005.docx]

**Supplementary References**

1. Earley KW, Haag JR, Pontes O, Opper K, Juehne T, et al. (2006) Gateway-compatible vectors for plant functional genomics and proteomics. Plant Journal 45: 616-629.

2. Traore SM, Zhao B (2011) A novel Gateway(R)-compatible binary vector allows direct selection of recombinant clones in Agrobacterium tumefaciens. Plant Methods 7: 42.

3. Xu B, Escamilla-Trevino LL, Noppadon S, Shen Z, Shen H, et al. (2011) Silencing of 4-coumarate:coenzyme A ligase in switchgrass leads to reduced lignin content and improved fermentable sugar yields for biofuel production. The New Phytologist 192: 611-625.
