# Supplementary material for: Overexpression of AtLOV1 in Switchgrass Alters Plant Architecture, Lignin Content, and Flowering Time
Source: PLoS One. 2012 Dec 26;7(12):e47399. doi: 10.1371/journal.pone.0047399 (PMC3530547; doi:10.1371/journal.pone.0047399)
Supplement: Table S1 — Primers used for PCR and RT-PCR. (DOCX) [file pone.0047399.s006.docx]

**Supplementary Table S1.** Primers used for PCR and RT-PCR.

| **Primer Sets** | **Primer Sequences (from 5’ to 3’)** |
| --- | --- |
| LOV1_SalRev | GTCGACCTTAAAAGGAATATTAGTATAG |
| LOV1_BamHFor | CACCGGATCCATGGCAATTGTATCCTCCACAAC |
| HYGRO_FOR | CAAACTGTGATGGACGACACCG |
| HYGRO_REV | TATATGCTCAACACATGAGCG |
| LOV1.2_FOR | CAAGACGATGATGAAACTGCC |
| AD_lov_276aa_RIFOR | ACGAATTCTCTGACGTTACCATTGCTCTAG |
| AD_lov_414aa_BamREV | ACGTCGACCTTAAAAGGAATATTAGTATAG |
| AD_lov_313aa_RIFOR | ACGAATTC ACTGCCATTGTTGACGATCTTC |
| AD_325aa_BamREV | ACGGATCCGTAGTTAACTAGTCTTTGAAG |
