# Supplementary material for: Overexpression of AtLOV1 in Switchgrass Alters Plant Architecture, Lignin Content, and Flowering Time
Source: PLoS One. 2012 Dec 26;7(12):e47399. doi: 10.1371/journal.pone.0047399 (PMC3530547; doi:10.1371/journal.pone.0047399)
Supplement: Table S2 — Primers used in qRT-PCR for the validation of ten selected genes identified by microarray analysis. (DOCX) [file pone.0047399.s007.docx]

**Supplementary Table S2.** Primers used in qRT-PCR for validation of ten selected genes identified by the microarray analysis.

| **probe set id** | **primers NAME** | **primer sequence** |  |
| --- | --- | --- | --- |
| AP13CTG21204_at | 21204_F | ACATCGCAGCCACAGGTTTG | |
| AP13CTG21204_at | 21204_R | CGGTGTTTGTACCGCAAGTG | |
| OTHSWCTG08878_s_at | 08878_F | GGCATCCATGCACTCTTCCT | |
| OTHSWCTG08878_s_at | 08878_R | CGACTCCTCATCCTCAGCATCTTAC | |
| AP13CTG06073_s_at | 06073_F | GAAGTGCATCTGCTTCAATCTGA | |
| AP13CTG06073_s_at | 06073_R | GTGGAAGTGATGACAGGATTGATC | |
| AP13ITG61387_at | 61387_F | TCGCGTATTCGCTTGTATAGTGA | |
| AP13ITG61387_at | 61387_R | ACGGCAGAGATCACACTAACCA | |
| KanlowCTG40909_s_at | 40909_F | ACCGCGGAGGTGAACTTCTG | |
| KanlowCTG40909_s_at | 40909_R | ATGCCCGAGTCAGTACCCAAAG | |
| AP13CTG01644_s_at | 01644_F | TCGCCACCTTCACATAACCA | |
| AP13CTG01644_s_at | 01644_R | GGGAGCACATCCTTGGATGA | |
| AP13ITG62738RC_at | 62738_F | CCTTCGGCATAATCGAAATCAAGA | |
| AP13ITG62738RC_at | 62738_R | CGGCTCGACAATCAGACATC | |
| AP13ITG40821_s_at | 40821_F | GCTGCAATGACGTTTACAGACA | |
| AP13ITG40821_s_at | 40821_R | CAGACGCAAACACTCACAAATCG | |
| AP13ITG76341_at | 76341_F | GCTGTGGTTACTGATGAATGGAAT | |
| AP13ITG76341_at | 76341_R | GGTCGATGGGACTGGTAGAAG | |
| KanlowCTG22618_s_at | 22618_F | ATCCGCCCGCGGAACCAT | |
| KanlowCTG22618_s_at | 22618_R | GGCCGACCGATTCTTGATGTAC | |
