# Supplementary material for: Overexpression of AtLOV1 in Switchgrass Alters Plant Architecture, Lignin Content, and Flowering Time
Source: PLoS One. 2012 Dec 26;7(12):e47399. doi: 10.1371/journal.pone.0047399 (PMC3530547; doi:10.1371/journal.pone.0047399)
Supplement: Table S3 — Differentially expressed genes in AtLOV1 transgenic switchgrass. | | | |, no information available; NA, homologs cannot be identified. (DOC) [file pone.0047399.s008.doc]

**Supplementary Table S3.** Differentially expressed genes in *AtLOV1* transgenic switchgrass plants. | | | |, no information available; NA, homologues cannot be identified.

| **Probeset_ID** | **Ratio of Gene Expression (Transgenic: WT)** | **Rice**  **Homolog** | **Arabidopsis Homolog** | **Description** |
| --- | --- | --- | --- | --- |
| OTHSWCTG07109_s_at | 0.18 | NA | AT3G23637 | AT3G23637 is a DVL family protein involved in plant development. Over-expression of some of this family proteins (eg.DVL1), results in pleiotropic phenotypes featured by shortened stature, rounder rosette leaves, clustered inflorescences, shortened pedicles, and siliques with pronged tips; DVL genes have overlapping function in the DVL gene family. |
| AP13CTG50047_at | 0.27 | NA | AT1G77131 | AT1G77131 is a pseudogene of PGSIP, glycogenin glucosyltransferase; and OsGGT relates to stress response. |
| KanlowCTG05419_at | 0.33 | Os02g32469 | NA | Uncharacterized protein; PRK12678, transcription termination factor Rho. |
| KanlowCTG17528_s_at | 0.33 | Os05g28740 | NA | Pfam00582, Universal stress protein family. The universal stress protein UspA is a small cytoplasmic bacterial protein whose expression is enhanced when the cell is exposed to stress agents. |
| KanlowCTG00502_at | 0.34 | Os02g01590 | AT1G62660 | Glycosyl hydrolases family 32 protein; Functions in: hydrolase activity, hydrolyzing O-glycosyl compounds; involved in: sucrose catabolic process, using beta-fructofuranosidase, carbohydrate metabolic process; Located in: vacuole; smart00640,Glycosyl hydrolases family 32, |AT1G62660,Galactose metabolism; Starch and sucrose metabolism; Metabolic pathways; Galactose metabolism. |
| AP13ITG74916_at | 0.35 | Os03g19470 | AT5G06700 | AT5G06700 encodes a member of the TBL (TRICHOME BIREFRINGENCE-LIKE) gene family containing a plant-specific DUF231 (domain of unknown function) domain. TBL gene family has 46 members, two of which (TBR/AT5G06700 and TBL3/AT5G01360) have been shown to be involved in the synthesis and deposition of secondary wall cellulose, presumably by influencing the esterification state of pectic polymers. |
| AP13ITG56631_s_at | 0.37 | Os08g04890 | AT5G44460 | AT5G44460 encodes calmodulin like protein; functions in calcium ion binding; involved in cadmium ion toxicity. |
| AP13CTG30372_s_at | 0.38 | Os11g34920 | AT3G07040 | AT3G07040 is RPS3 (RPM1), an NB-LRR gene, conferring disease resistance to *Pseudomonas syringae pv. tomato* contain *avrB* and *avrRpm1.* |
| AP13CTG21204_at | 0.38 | Os06g35940 | AT1G65480 | The FT homolog; FT, together with LFY, promotes flowering and is antagonistic with its homologous gene. late flowering phenotype under long-day conditions (40.6 leaves versus 15.0 leaves for wild type); late flowering; strong influence of short days on flowering time; small but significant effect of vernalization on flowering time; narrow leaves, increased number of cauline leaves; flowering occurs about 13 days later than wild type. |
| AP13ITG36831_at | 0.39 | NA | NA | | | | | |

| KanlowCTG23086_s_at | 0.40 | Os11g12740 | AT1G33440 | Protein has transporter activity; Arabidopsis homolog has oligopeptide transport function, and responds to the nematode stress. Pfam00854, POT family. The POT (proton-dependent oligopeptide transport) family all appear to be proton dependent transporters. |
| --- | --- | --- | --- | --- |
| KanlowCTG16502_s_at | 0.42 | Os11g26790 | NA | Possibly a grass-specific dehydrin; pfam00257, Dehydrin. |
| KanlowCTG12183_s_at | 0.42 |  | AT1G20450 | AT1G20450 involved in cold acclimation, regulation of seed germination, response to abscisic acid stimulus, response to cold, response to stress, response to water, response to water deprivation; colocalizes with actin cytoskeleton; functions in actin binding. |
| OTHSWCTG08878_s_at | 0.42 | Os05g38680 | AT3G55240 | Over-expression of AT3G55240 leads to PEL (Pseudo-Etiolation in Light) phenotype; Pfam09713, Plant protein 1589 of unknown function (A_thal_3526). |
| KanlowCTG33856_s_at | 0.42 | Os03g61150 | AT1G19530 | AT1G19530, involved in N-terminal protein myristoylation, anaerobic F16 respiration; expressed in leaf apex, inflorescence meristerm, hypocotyl, root, flower; PHA03307, transcriptional regulator ICP4. |
| AP13CTG72550_at | 0.42 | Os03g28300 | AT1G79570 | Arabidopsis homologs involved in pollen germination. pfam07714, Protein tyrosine kinase, |SERINE/THREONINE PROTEIN KINASE. |
| AP13ITG53107_at | 0.43 | Os03g03510 | AT1G01140 | Catalytic domain of the Protein Serine/Threonine Kinase, STKs catalyze the transfer of the gamma-phosphoryl group from ATP to potassium ion cellular response to potassium ion starvation, regulation of potassium ion transport, response to response to water deprivation, somatal movement, potassium ion starvation, cold, mannitol stimulus, salt and wounding; serine/threonine kinase activity; involved in signal transduction pathway; locates at cytoplasm, cytosol, nucleus, plasma membrane; serine/threonine residues on protein substrates. PKA is present ubiquitously in cells and interacts with many different downstream targets. It plays a role in the regulation of diverse processes such as growth, development, memory, metabolism, gene expression, immunity, and lipolysis. |
| AP13ITG74064_at | 0.43 | Os09g35810 | AT1G01225 | Unknown function; |NC Domain-containing protein. |
| AP13ITG39221_s_at | 0.44 | Os01g36580 | AT5G64700 | Auxin-induced protein 5NG4, response to auxin stimulus; Arabidopsis homolog is close to walls thin 1 (WAT1), an arabidopsis homolog of *Medicago truncatula* Nodulin21, is a tonoplast-localized protein required for secondary wall formation in fibers. Analysis of wat1 mutants revealed two cell wall-related phenotypes in stems: a defect in cell elongation, resulting in a dwarfed habit and little to no secondary cell walls in fibers. |
| AP13CTG27860_at | 0.45 | Os06g48020 | AT4G30170 | Horseradish peroxidase and related secretory plant peroxidases. Class III peroxidases are found in the extracellular space or in the vacuole in plants where they have been implicated in hydrogen peroxide detoxification, auxin catabolism and lignin biosynthesis, and stress response. Class III peroxidases contain four conserved disulphide bridges and two conserved calcium binding sites. |AT4G30170, Phenylpropanoid biosynthesis; |Os06g48020.1 Phenylpropanoid biosynthesis. |
|  | | | | |

| OTHSWCTG14473_s_at | 0.45 | NA | NA | | | | | |
| --- | --- | --- | --- | --- |
| KanlowCTG14571_s_at | 0.46 | Os01g48444 | AT2G22670 | Pfam02309, AUX/IAA family. Arabidopsis homolog is IAA8; Transcription of the AUX/IAA family of genes is rapidly induced by the plant hormone auxin. Some members of this family are longer and contain an N-terminal DNA binding domain. The function of this region is uncertain. |
| KanlowCTG11191_at | 0.48 | NA | NA | | | | | |
| AP13CTG03030_at | 0.48 | Os02g05660 | AT1G55150 | COG0513, Superfamily II DNA and RNA helicases [DNA replication, recombination, and repair / Transcription / Translation, ribosomal structure and biogenesis] |AT1G55150, Spliceosome|Os02g05660.1, Spliceosome. |
| AP13ITG66127_at | 0.48 | Os03g55030 | AT5G05870 | UDP-GLUCOSYLTRANSFERASE|AT5G05870, Zeatin biosynthesis. |
| KanlowCTG40909_s_at | 0.48 | Os04g33280 | AT5G47860 | Unknown function |
| AP13ITG76411RC_at | 0.48 | Os05g43610 | AT4G21070 | BRCA1 C Terminus (BRCT) domain. The BRCT domain is found predominantly in proteins involved in cell cycle checkpoint functions responsive to DNA damage. |
| OTHSWCTG14744_at | 0.49 | NA | NA | | | | | |
| AlamoCTG08260_s_at | 0.49 | Os04g17100 | [At5g66110.1](http://greenphyl.cirad.fr/cgi-bin/sequence.cgi?search=At5g66110.1) | Unknown function. |
| AP13ITG76439_at | 0.49 | Os01g20830 | NA | Uncharacterized protein. |
| OTHSWCTG17883_at | 0.49 | Os05g40820 | AT3G53020 | Ribosomal protein L24e. |
| AP13ITG67117_at | 0.50 | Os06g35060 | NA | COPPER TRANSPORT PROTEIN ATOX1 (METAL TRANSPORT PROTEIN ATX1). |
| AP13CTG25639_s_at | 0.50 | Os11g41500 | NA | Unknown function. |
| KanlowCTG23960_s_at | 0.50 | Os07g44290 | AT3G17510 | Serine/Threonine protein kinases, catalytic domain. Phosphotransferases. Serine or threonine-specific kinase subfamily. |
| KanlowCTG21211_at | 0.50 | Os01g01340 | AT3G26740 | Light regulated protein Lir1. Lir1 mRNA accumulates in the light, reaching maximum and minimum steady-state levels at the end of the light and dark period, respectively. Plants germinated in the dark have very low levels of lir1 mRNA, whereas plants germinated in continuous light express lir1 at an intermediate but constant level. It is thought that lir1 expression is controlled by light and a circadian clock. The exact function of this family is unclear. |
| KanlowCTG13627_s_at | 2.01 | Os03g56270 | AT5G65700 | Leucine-rich repeat receptor-like protein kinase. |

| KanlowCTG05958_s_at | 2.01 | Os07g28480 | AT1G17180 | GST_C family, Class Tau subfamily; The plant-specific class Tau GST subfamily has undergone extensive gene duplication. The Arabidopsis and Oryza genomes contain 28 and 40 Tau GSTs, respectively. They are primarily responsible for herbicide detoxification together with class Phi GSTs, showing class specificity in substrate preference. Tau enzymes are highly efficient in detoxifying diphenylether and aryloxyphenoxypropionate herbicides. In addition, Tau GSTs play important roles in intracellular signaling, biosynthesis of anthocyanin, responses to soil stresses and responses to auxin and cytokinin hormones. |
| --- | --- | --- | --- | --- |
| AP13ITG69649RC_at | 2.01 | Os06g30640 | AT2G45570 | Flavonoid 3'-monooxygenase, |
| AP13ITG60851-RC_at | 2.02 | Os10g38140 | AT1G10370 | GST_C family, Class Tau subfamily; The plant-specific class Tau GST subfamily has undergone extensive gene duplication. The Arabidopsis and Oryza genomes contain 28 and 40 Tau GSTs, respectively. They are primarily responsible for herbicide detoxification together with class Phi GSTs, showing class specificity in substrate preference. Tau enzymes are highly efficient in detoxifying diphenylether and aryloxyphenoxypropionate herbicides. In addition, Tau GSTs play important roles in intracellular signalling, biosynthesis of anthocyanin, responses to soil stresses and responses to auxin and cytokinin hormones. |
| AP13ITG40032_s_at | 2.03 | Os03g09270 | AT3G49600 | Unknown function. |
| KanlowCTG15754RC_s_at | 2.04 | Os03g52010 | AT1G27480 | Lecithin:cholesterol acyltransferase. Lecithin:cholesterol acyltransferase (LACT) is involved in extracellular metabolism of plasma lipoproteins, including cholesterol. |
| AP13ITG57916_at | 2.04 | NA | NA | | | | | |
| OTHSWCTG01972_s_at | 2.05 | Os01g29330 | AT4G24310 | Protein of unknown function (DUF679). This family contains several uncharacterized plant proteins. |
| AP13ITG38627_at | 2.05 | Os01g50200 | AT3G16520 | PLN00164,glucosyltransferase; |UDP-Glucosyltransferase, |AT3G50740, Phenylpropanoid biosynthesis. |
| KanlowCTG06676_s_at | 2.05 | Os11g03240 | AT1G33110 | Putative efflux protein, MATE family. The MATE family consists of probable efflux proteins including a functionally characterized multi drug efflux system from *Vibrio parahaemolyticus*, a putative ethionine resistance protein of *Saccharomyces cerevisiae*, and the functionally uncharacterized DNA damage-inducible protein F (DinF) of *E. coli*. |
| AP13CTG14683_at | 2.05 | Os04g44530 | AT1G73390 | BRO1-like domain. This domain is found in number proteins including Rhophilin and BRO1. |
| KanlowCTG00810_at | 2.06 | Os07g26630 | AT3G53420 | Major intrinsic protein. MIP (Major Intrinsic Protein) family proteins exhibit essentially two distinct types of channel properties: (1) specific water transport by the aquaporins, and (2) small neutral solutes transport, such as glycerol by the glycerol facilitators. |
| KanlowCTG16521_at | 2.09 | Os06g08830 | AT2G36750 | UDP-glucosyltransferase family protein |AT2G36750, Zeatin biosynthesis. |

| KanlowCTG04653_s_at | 2.10 | Os03g08330 | AT1G19180 | Tify domain. This short possible domain is found in a variety of plant transcription factors that contain GATA domains as well as other motifs. Although previously known as the Zim domain this is now called the tify domain after its most conserved amino acids. TIFY proteins can be further classified into two groups depending on the presence (group I) or absence (group II) of a C2C2-GATA domain. Functional annotation of these proteins is still poor, but several screens revealed a link between TIFY proteins of group II and jasmonic acid-related stress response. |
| --- | --- | --- | --- | --- |
| AP13CTG01644_s_at | 2.11 | Os01g60700 | AT3G08760 | Catalytic domain of Protein Tyrosine Kinases. Protein Tyrosine Kinase (PTK) family, catalytic domain. |
| AP13CTG25169_at | 2.11 | Os05g02310 | AT3G53620 | PLN02373, inorganic pyrophosphatase, |AT3G53620,Oxidative phosphorylation|Os05g02310.1|SORBI_09g001530,Oxidative phosphorylation. |
| KanlowCTG07634_at | 2.11 | Os12g31000 | AT1G78160 | Pumilio-family RNA binding domain. These proteins function as translational repressors in early embryonic development. |
| AP13ITG42359_s_at | 2.12 | 08g015360 | AT5G20850 | DNA REPAIR PROTEIN RAD51 HOMOLOG 3, R51H3; |AT5G20850,Homologous recombination; |Os01g39630.1; |SORBI_08g015360, Homologous recombination related protein. |
| KanlowCTG47379_s_at | 2.13 | Os04g33590 | AT2G36290 | *alpha/beta* hydrolase fold. This catalytic domain is found in a very wide range of enzymes. |
| OTHSWSLT37179_s_at | 2.13 | NA | AT5G55240 | | | | | |
| AP13ITG62738RC_at | 2.14 | Os07g04930 | [At4g38080.1](http://greenphyl.cirad.fr/cgi-bin/sequence.cgi?search=At4g38080.1) | DNA polymerase III subunits gamma and tau. |
| AP13ITG69404_at | 2.17 | Os06g09240 | AT5G17050 | UDP-glucoronosyl and UDP-glucosyl transferase; |OS06G0192100 |AT5G17050, Flavone and flavonol biosynthesis; Metabolic pathways; Biosynthesis of secondary metabolites. |
| AP13ITG43449_at | 2.17 | NA | NA | | | | | |
| AP13CTG04833_s_at | 2.18 | Os08g14570 | AT4G30210 | NADPH cytochrome p450 reductase (CYPOR) serves as an electron donor in several oxygenase systems and is a component of nitric oxide synthases and methionine synthase reductases. |
| AP13ITG59202_at | 2.19 | Os06g29730 | [AT1G64450.](http://www.arabidopsis.org/servlets/TairObject?type=locus&name=AT1G64450) | Unknown function. |
| KanlowCTG08996RC_s_at | 2.20 | Os03g22790 | [At5g18670.1](http://greenphyl.cirad.fr/cgi-bin/sequence.cgi?search=At5g18670.1) | *beta*-amylase; |AT4G17090,Starch and sucrose metabolism; Metabolic pathways|Os03g22790.1|. |
| KanlowCTG20184_at | 2.23 | Os11g38810 | AT1G67070 | Mannose-6-phosphate isomerase, |Os11g38810.1;13111|SORBI_05g023560, Fructose and mannose metabolism; Amino sugar and nucleotide sugar metabolism; Metabolic pathways; Biosynthesis of secondary metabolites. |
| AP13ITG69747_s_at | 2.26 | Os06g42560 | AT5G38530 | Tryptophan synthase subunit beta; |AT5G38530,Glycine, serine and threonine metabolism; Phenylalanine, tyrosine and tryptophan biosynthesis; Metabolic pathways; Biosynthesis of secondary metabolites|Os06g42560.2, Glycine, serine and threonine metabolism; Phenylalanine, tyrosine and tryptophan biosynthesis; Metabolic pathways; Biosynthesis of secondary metabolites. |
| KanlowCTG18753_s_at | 2.27 | Os02g49870 | [At4g01150.1](http://greenphyl.cirad.fr/cgi-bin/sequence.cgi?search=At4g01150.1) | PLN02777, photosystem I |
| AP13CTG11797_s_at | 2.27 | Os01g60830 | At3g56290 | Unknown function |
| AP13CTG23828-RC_at | 2.30 | Os04g46440 | NA | DNA-binding domain in plant proteins such as APETALA2 and EREBPs. |

| AP13ITG66354_at | 2.32 | Os12g04424 | NA | Strictosidine synthase. Strictosidine synthase is a key enzyme in alkaloid biosynthesis. |
| --- | --- | --- | --- | --- |
| KanlowCTG12631_at | 2.33 | Os04g20230 | AT5G55940 | Unknown function |
| AP13CTG27072_s_at | 2.33 | Os05g08770 | NA | Unknown function |
| AP13ITG55695_at | 2.33 | Os01g45200 | AT1G15950 | Cinnamoyl-CoA reductase, |CINNAMOYL-COA REDUCTASE, |AT1G15950, Phenylpropanoid biosynthesis; Metabolic pathways; Biosynthesis of secondary metabolites; |Os01g45200.1|SORBI_07g021680,Phenylpropanoid biosynthesis; Metabolic pathways; Biosynthesis of secondary metabolites. |
| AP13ITG60331-RC_at | 2.34 | NA | NA | | | | | |
| AP13ITG35680_at | 2.36 | Os01g60830 | [At3g56290.1](http://greenphyl.cirad.fr/cgi-bin/sequence.cgi?search=At3g56290.1) | Unknown function |
| AP13ITG40821_s_at | 2.39 | Os01g4273 | [At1g28600.1](http://greenphyl.cirad.fr/cgi-bin/sequence.cgi?search=At1g28600.1) | SGNH_plant_lipase_like, a plant specific subfamily of the SGNH-family of hydrolases, a diverse family of lipases and esterases. |LATERAL SIGNALING TARGET PROTEIN. |
| AP13ITG76341_at | 2.43 | Os04g35020.1 | [At2g32540.1](http://greenphyl.cirad.fr/cgi-bin/sequence.cgi?search=At2g32540.1) | cellulose synthase, |CELLULOSE SYNTHASE-LIKE PROTEIN. |
| AP13CTG15471_s_at | 2.51 | Os01g16030.4 | [At1g10630.1](http://greenphyl.cirad.fr/cgi-bin/sequence.cgi?search=At1g10630.1) | ARF family protein; Provisional, |Family not named. |
| OTHSWCTG10168_at | 2.53 | NA | NA | | | | | |
| AP13CTG01028_at | 2.55 | Os06g14324.1 | NA | Caleosin related protein. This family contains plant proteins related to caleosin. Caleosins contain calcium-binding domains and have an oleosin-like association with lipid bodies. Caleosins are present at relatively low levels and are mainly bound to microsomal membrane fractions at the early stages of seed development. As the seeds mature, overall levels of caleosins increased dramatically and they were associated almost exclusively with storage lipid bodies. |
| KanlowCTG32425_s_at | 2.55 | Os06g39960.1 | NA | Basic region leucine zipper. Unknown function. |
| KanlowCTG11482_s_at | 2.56 | Os02g44200.1 | NA | Predicted thioesterase [General function prediction only] |
| AP13ITG43545_s_at | 2.59 | Os02g10860.1 | [At5g11260.1](http://greenphyl.cirad.fr/cgi-bin/sequence.cgi?search=At5g11260.1) | Basic region leucine zipper, Unknown function. |
| KanlowCTG36752_s_at | 2.77 | Os10g40030.8 | NA | oxidoreductase; Unknown function. |
| OTHSWCTG07314_at | 2.78 | Os06g39960.1 | NA | Basic region leucine zipper. Unknown function. |
| AP13ITG57635_at | 2.92 | Os08g08850.1 | [At4g27030.1](http://greenphyl.cirad.fr/cgi-bin/sequence.cgi?search=At4g27030.1) | Kua-ubiquitin conjugating enzyme hybrid localization domain. |
| AP13CTG27675_s_at | 3.03 | Os03g04470.1 | [At2g36885.1](http://greenphyl.cirad.fr/cgi-bin/sequence.cgi?search=At2g36885.1) | Transcription termination factor Rho. |
| KanlowCTG22618_s_at | 3.11 | Os02g43640.1 | NA | *alpha*-ketoglutarate decarboxylase. |
| AP13ITG43544_s_at | 3.14 | Os02g10860.1 | [At5g11260.1](http://greenphyl.cirad.fr/cgi-bin/sequence.cgi?search=At5g11260.1) | Basic region leucine zipper, Unknown function. |

| AP13ITG41313_at | 3.68 | NA | NA | | | | | |
| --- | --- | --- | --- | --- |
| AP13CTG05998_at | 3.81 | Os10g40020.3 | NA | Oxidoreductase; |Family not named. |
| AP13CTG22352_at | 3.97 | NA | NA | | | | | |
| KanlowCTG39887_at | 5.01 | Os05g33310.1 | AT4G02940 | AT4G02940.1 belongs to pfam03171, 2OG-Fe(II) oxygenase superfamily. |
